# STAT2 hinders STING intracellular trafficking and reshapes its activation in response to DNA damage

**DOI:** 10.1073/pnas.2216953120

**Published:** 2023-04-10

**Authors:** Chenyao Wang, Jing Nan, Elise Holvey-Bates, Xing Chen, Samantha Wightman, Muhammad-Bilal Latif, Junjie Zhao, Xiaoxia Li, Ganes C. Sen, George R. Stark, Yuxin Wang

**Affiliations:** ^a^Department of Inflammation and Immunity, Lerner Research Institute, The Cleveland Clinic Foundation, Cleveland, OH 44195; ^b^Department of Medicine, Mayo Clinic College of Medicine and Science, Mayo Clinic, Rochester, MN 55905; ^c^Institute of Cancer Biology and Drug Screening, School of Life Sciences, Lanzhou University, Lanzhou, Gansu 730000, China; ^d^Department of Cancer Biology, Lerner Research Institute, The Cleveland Clinic Foundation, Cleveland, OH 44195; ^e^Pathology Advanced Translational Research Unit, Department of Pathology and Laboratory Medicine, School of Medicine, Emory University, Atlanta, GA 30322

**Keywords:** STAT2, STING, DNA damage

## Abstract

In cancer cells, endogenous or therapy-induced DNA damage leads to the abnormal presence of DNA in the cytoplasm, which triggers the activation of cGAS (cyclic GMP–AMP synthase) and STING (stimulator of interferon genes). STAT2 suppresses the cGAMP-induced expression of IRF3-dependent genes by binding to STING, blocking its intracellular trafficking, which is essential for the full response to STING activation. STAT2 reshapes STING signaling by inhibiting the induction of IRF3-dependent, but not NF-κB–dependent genes. This noncanonical activity of STAT2 is regulated independently of its tyrosine phosphorylation but does depend on the phosphorylation of threonine 404, which promotes the formation of a STAT2:STING complex that keeps STING bound to the endoplasmic reticulum (ER) and increases resistance to DNA damage. We conclude that STAT2 is a key negative intracellular regulator of STING, a function that is quite distinct from its function as a transcription factor.

## Significance

Therapeutic failure is a major problem for cancer patients. Chemotherapy often induces DNA damage or arrest of DNA synthesis, leading to the abnormal presence of DNA in the cytoplasm, which activates the cGAS–STING pathway. STAT2 binds to STING, modulating its function by inhibiting the expression of IRF3-dependent antitumor genes, but not of NF-κB–dependent protumor genes, thus helping cancer cells to resist DNA damage. This function of STAT2 is dependent on its phosphorylation on T404, a high level of which is seen in about 25% of human lung cancer specimens. Our data reveal a mechanism, quite distinct from its regulation of transcription, through which STAT2 reshapes STING activation and promotes malignancy.

Stimulator of interferon genes (STING) plays a crucial role in regulating the activation of innate and adaptive immunity. By directly binding to cyclic dinucleotides (CDNs), STING induces the expression of host defense genes whose products mediate essential antimicrobial and antitumor immunity (1–3). CDNs induce conformational changes of STING, which lead to its departure from the endoplasmic reticulum (ER) (4). This intracellular trafficking is essential for the full response to STING activation (4). STING migrates from the ER to ERGIC (ER-Golgi intermediate compartment) and the Golgi body, where it recruits and activates TBK1, which phosphorylates S366 of STING (5, 6). Phosphorylated S366 is a docking site for IRF3 and is essential for the subsequent induction of Interferons (IFNs) (7, 8). Our previous work demonstrated that IRF3 binds to phosphorylated STING in the late endosomal compartment, where TBK1 phosphorylates and activates IRF3 (9).

Additional proteins regulate the trafficking of STING. STIM1 (stromal interaction molecule 1), a transmembrane protein that mediates Ca^2+^ influx, inhibits STING activation by causing its retention in the ER (10, 11). In contrast, iRhom2 (inactive rhomboid protein 2) and STEEP interact with STING and facilitate its trafficking from the ER and its functions (12, 13). In the present study, we show that STAT2 is a negative regulator of STING intracellular trafficking, which is essential for the full response to its activation. Furthermore, we show that E316 of STING and the phosphorylation of T404 in the DNA-binding domain (DBD) of STAT2 are both required to form the complex with STAT2 that blocks STING trafficking.

It is well known that STAT2 helps to transduce type I IFN-dependent signals when its tyrosine residue Y690 is phosphorylated (14). STAT2 also contributes to tumorigenesis in various types of cancer (15). We have shown that elevated levels of tyrosine-unphosphorylated STAT2 and IRF9 enhance the expression of a subset of NF-κB–dependent genes, including IL-6 (16, 17). Importantly, a high level of STAT2 drives a gene signature that is associated with therapeutic resistance and poor prognosis (18, 19). Here, we report a protumor function of STAT2 that inhibits the activation of STING in cancer cells. STING activation leads in turn to the activation of IRF3 and NF-κB (20). The antitumor activities of STING depend primarily on IRF3 activation (21, 22). STING senses therapy-induced or endogenous DNA damage and consequently stimulates antitumor immune responses, including the recruitment and activation of cytotoxic T-cells (23, 24). STING also activates NF-κB, which promotes progression and resistance to therapy by inducing the production of inflammatory cytokines (25), including IL-6, which is required for the survival of cancer cells following genotoxic treatments (26). Moreover, we found that STAT2 reshapes STING signaling by suppressing the induction of IRF3-dependent but not NF-κB–dependent genes. The phosphorylation of STAT2 on T404 inhibits the DNA damage response that is induced by exposure of lung cancer cells to cisplatin and may correlate with the infiltration of fewer CD8^+^ T cells in lung cancers. Our data indicate that tumor cells reshape and evade STING-mediated antitumor effects by manipulating the expression level and T404 phosphorylation of STAT2.

## Results

### STAT2 Inhibits IFN-β Induction in Response to STING Ligands.

**Fig. 1.**
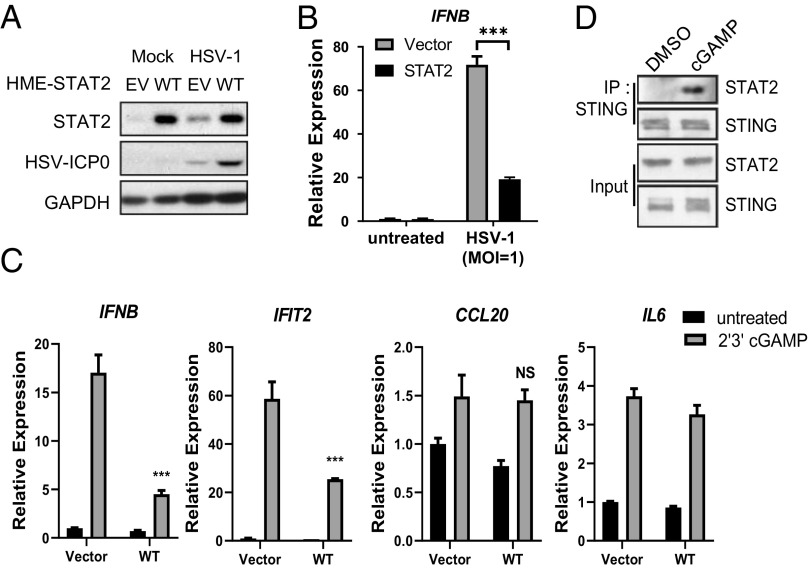
STAT2 interacts with STING and inhibits induction of IFN-β. (*A*) HMEs (human mammary epithelial cells) stably expressing STAT2 were infected with HSV-1 (MOI = 1) for 20 h. Whole-cell lysates were analyzed by the Western blot method. (*B*) HMEs stably expressing STAT2 were infected with HSV-1 (MOI = 1) for 6 h. Total RNAs were analyzed by qPCR. (*C*) HMEs overexpressing parental or STAT2 were stimulated with 2′3′cGAMP (8 μg/mL) for 4 h, followed by RT-PCR analysis. (*D*) IP analysis of endogenous STAT2–STING interaction in HT1080 cells treated with 8 µg/mL cGAMP for 3 h.

Mammalian cells detect DNA viruses through the cGAS–STING pathway (3). cGAS recognizes cytoplasmic DNA and generates 2′, 3′-Cyclic guanosine monophosphate–adenosine monophosphate (cGAMP), an intracellular second messenger that directly binds to and activates STING to induce the transcription of IRF3- and NF-κB–dependent genes, whose products elicit antiviral and proinflammatory responses (20, 27, 28). We find that high expression of STAT2 compromises the antiviral response to Herpes Simplex Virus 1 (HSV-1) (Fig. 1*A*), a DNA virus, due to insufficient production of IFN-β (Fig. 1*B*). We propose that high expression of STAT2 compromises the antiviral defense against HSV by inhibiting STING activation. When cells expressing a high level of STAT2 were treated with 2′, 3′-cGAMP, the induction of IFN-β expression was substantially inhibited (Fig. 1*C*). Interestingly, STAT2 preferentially inhibits the expression of IRF3-dependent genes but not NF-κB–dependent genes (Fig. 1*C*). Similar results were observed with Calu-1 (nonsmall cell lung cancer cells, *SI Appendix*, Fig. S1*A*), and THP1 (immortalized human monocytes, *SI Appendix*, Fig. S1*B*). By directly binding to microbial (CDNs), STING also connects microbial sensing with the induction of multiple host defense genes (29). Consistently, the STING ligands cdi-cyclic dimeric guanosine monophosphate (GMP) and 2′, 3′-cGAMP gave similar results (*SI Appendix*, Fig. S1*C*). In summary, a function of STAT2 inhibits STING activation in response to various ligands.

### STAT2 Interacts with STING and Inhibits STING-Dependent Signaling.

STAT2 binds to STING upon stimulation with 2′, 3′-cGAMP (Fig. 1*D*), stimulating an investigation of how STAT2 interacts with STING and inhibits its activation. Using a set of mGST-fusion proteins with different STING truncations, we detected direct interaction between residues 265 to 379 of STING and STAT2 (*SI Appendix*, Fig. S2*A*). Analysis of several deletions within the 265 to 379 region revealed that residues 309 to 316 are necessary for binding to STAT2 (*SI Appendix*, Fig. S2 *B* and *C*).

Furthermore, the single STING mutation E316A disrupts the interaction (*SI Appendix*, Fig. S2*D*). Critically, E316 is highly conserved among species (*SI Appendix*, Fig. S2*E*). The negative charge of E316 is critical for the intramolecular interaction between the CDN-binding domain and the C-terminal tail, which keeps STING in a dormant state (30). Loss of this negative charge might change its conformation and switch STING to a hyper-active state (31). We made HT1080 cells deficient in STING using CRISPR-Cas9 and then restored STING expression with either wild-type (WT) or the E316A mutant. Upon HSV-1 infection, E316A-STING drove a more robust induction of the expression of *IFNB*, an IRF3-dependent gene, but not of *CCL20*, an NF-κB–dependent gene (*SI Appendix*, Fig. S2*F*). Consequently, HSV replication is lower in cells expressing E316A-STING than WT-STING (*SI Appendix*, Fig. S2*G*). This altered gene expression pattern repeats the finding that STAT2 specifically inhibits IRF3-dependent gene induction. Our discovery agrees with previous findings indicating that E316 is essential to enable STING to interact with STAT2, thus strengthening the dormant state of STING. Therefore, STAT2 is a vital negative regulator of STING-dependent signaling, especially with regard to IFN-β production and antiviral activity.

### STAT2 Impedes STING Trafficking from the ER.

**Fig. 2.**
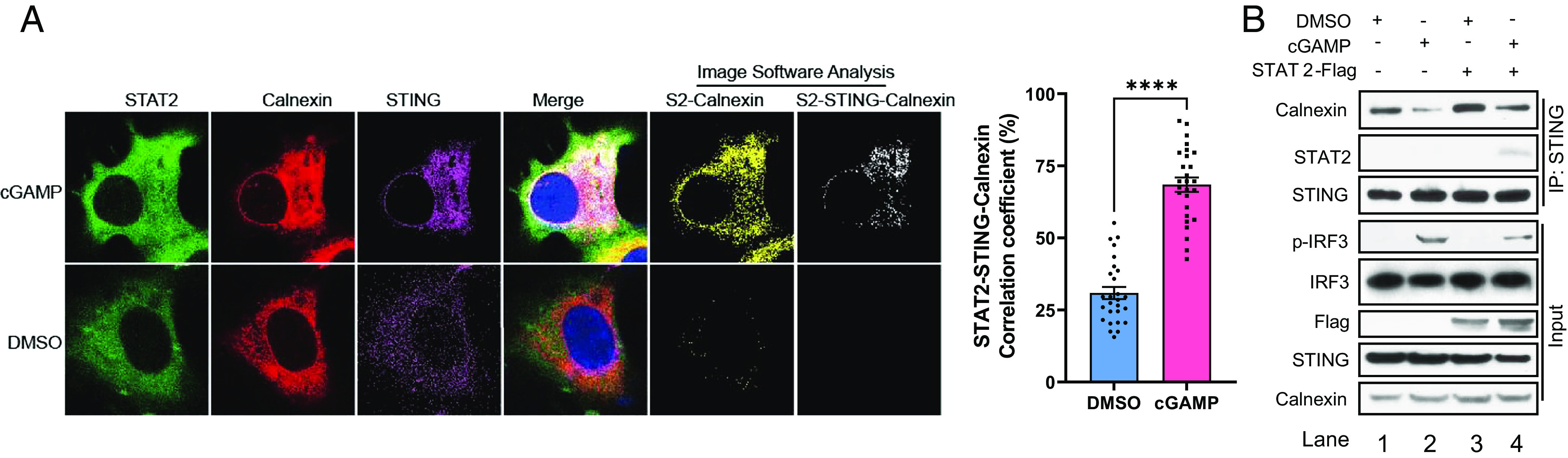
STAT2 impedes STING trafficking from ER. (*A*) Calu-1 cells were transfected with STAT2–GFP (green) and STING–HA for 24 h and treated with cGAMP for 1 h. The cells were fixed and stained with calnexin antibody (red) and HA antibody (purple). Imaging data from confocal fluorescence microscopy were analyzed by Fuji software to reveal colocalization, shown as white dots. (*B*) THP1 cells over expressing vector or STAT2-flag (WT) were stimulated with 2′3′cGAMP (8 μg/mL) for 3 h, followed IP analysis of STAT2–STING–calnexin interactions.

The ligand-induced formation of STING dimers and oligomers enables full activation of downstream signaling. STING translocates from the ER through the ER–Golgi and Golgi-to-post-Golgi compartments (4, 5). During its translocation, STING mediates the activation of several kinases, including TANK-binding kinase 1 (TBK1) and IκB Kinases (IKKs), which mediate downstream transcription. Since intracellular trafficking is essential for complete STING activation (4), we investigated whether STAT2 colocalizes with STING, using Bimolecular Fluorescence Complementation. STAT2 interacts with STING primarily in the cytosol, especially in the ER (*SI Appendix*, Fig. S3*A*), where STING resides in the absence of stimulation (3). Interestingly, confocal microscopy revealed that cGAMP enhances the translocation of STAT2 to the ER in Calu-1 cells (Fig. 2*A* and *SI Appendix*, Fig. S3*B*) and primary human peripheral blood mononuclear cells (*SI Appendix*, Fig. S3*C*).

To confirm that STAT2 translocates to the ER upon treatment with a STING ligand, Co-IP experiments were performed, showing that both STING and STAT2 bind to calnexin, a chaperone protein located on the ER membrane (*SI Appendix*, Fig. S3*D*). We speculate that STAT2 does not enter the ER lumen and may interact with the C-terminus of calnexin, which faces the cytoplasm, while its N-terminus faces the ER lumen (32). Furthermore, the CCD domain of STAT2 is needed for this interaction (*SI Appendix*, Fig. S3*E*). In THP1 cells, treatment with cGAMP decreased the association of STING with calnexin, revealing the departure of STING from the ER (Fig. 2*B*, comparing lanes 1 and 2). Exogenously increased expression of STAT2 prevents the relocation of STING from the ER, as shown by a strengthened interaction between STING and calnexin (Fig. 2*B*, comparing lanes 2 and 4). These data reveal that STAT2 inhibits STING activation by suppressing its trafficking.

### Phosphorylation of T404 Drives the Inhibition of STING by STAT2.

**Fig. 3.**
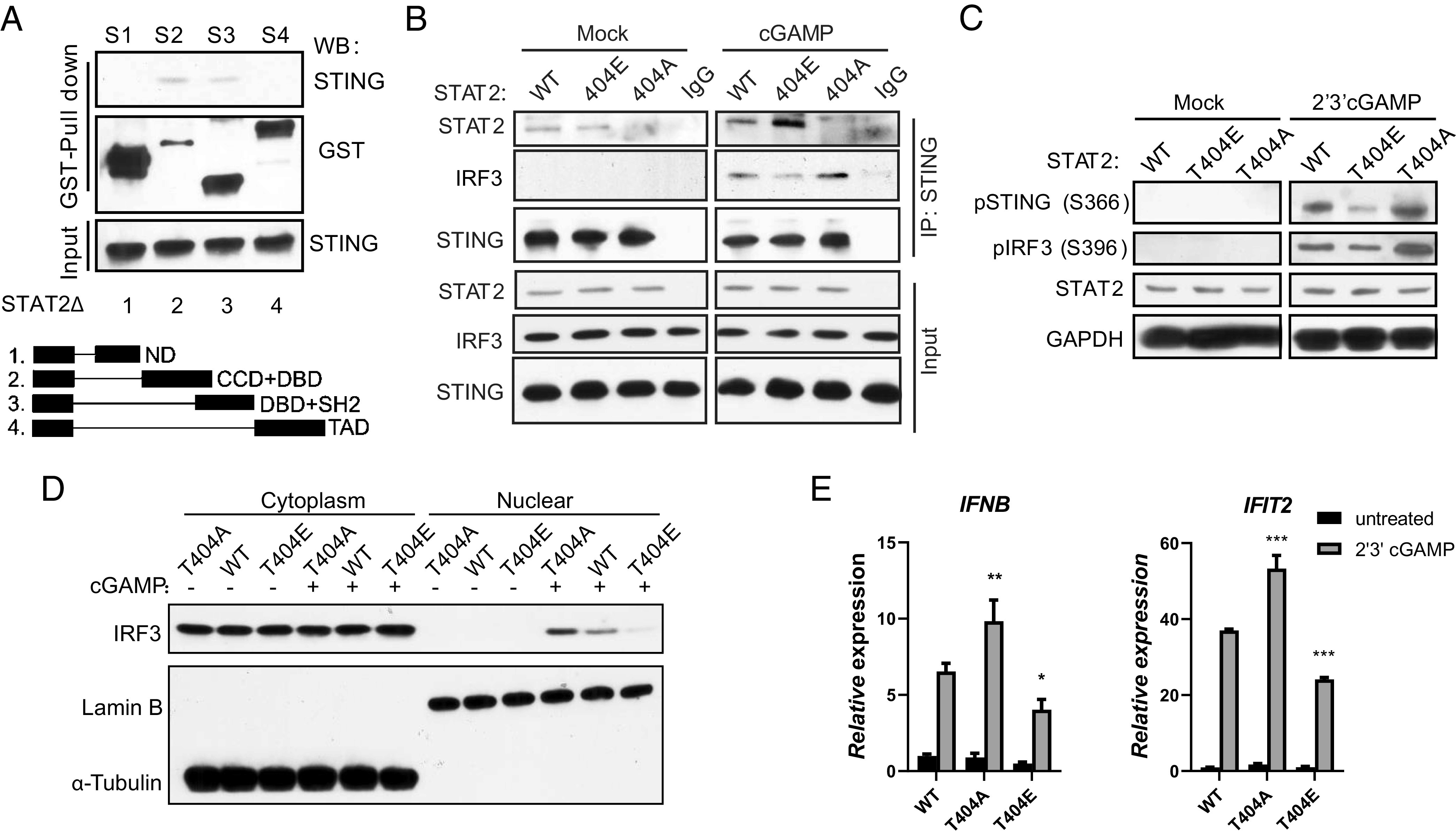
Phosphorylation of T404 drives STAT2 inhibition of STING. (*A*) GST-pull-down assay using HEK293T cells transfected with GST-tagged truncations of STAT2. N-terminal domain, CCD: coil-coiled domain, DBD, trans-activation domain; (*B*) STAT2-deficient Calu-1 cells restored with WT, T404A, or T404E STAT2 were stimulated with 2′3′cGAMP (8 μg/mL) for 3 h, followed by IP analysis of the STAT2–STING–IRF3 interaction. (*C*) STAT2-deficient Calu-1 cells restored with WT, T404A, or T404E STAT2 were stimulated with 2′3′cGAMP (8 μg/mL) for 3 h, followed by Western analysis. (*D*) STAT2-deficient Calu-1 cells restored with WT, T404A, or T404E STAT2 were stimulated with 2′3′cGAMP (8 μg/mL) for 3 h, followed by nuclear fractionation and analysis by the Western method. (*E*) STAT2-deficient Calu-1 cells restored with WT, T404A, or T404E STAT2 were stimulated with 2′3′cGAMP (8 μg/mL) for 4 h, followed by RT-PCR analysis.

To determine whether the inhibition of STING activation depends on the phosphorylation of STAT2 on Y690, we expressed Y690F or WT STAT2 in STAT2-deficient Calu-1 cells, finding that the ability of STAT2 to blunt gene expression in response to 2′, 3′-cGAMP is independent of tyrosine phosphorylation (*SI Appendix*, Fig. S4). By studying deletions of STAT2, we found that the DBD of STAT2 is required for the STAT2:STING complex to form (Fig. 3*A*). IKKi phosphorylates STAT2 on T404, which is in the DBD (33), and IKKi is activated in response to STING activation (34). Stimulation with 2′, 3′-cGAMP induced T404 phosphorylation (*SI Appendix*, Fig. S5*A*). To determine whether T404 phosphorylation of STAT2 is critical for its interaction with STING, WT, T404A, or T404E, WT STAT2 or the two mutants were expressed in STAT2-deficient Calu-1 cells. Upon treatment with cGAMP, the T404A mutation, which prevents phosphorylation, blocked the ability to bind to STING. In contrast, the phospho-mimetic T404E mutation led to stronger binding (Fig. 3*B*). In addition, STING recruited less IRF3 in cells expressing T404E-STAT2 (Fig. 3*B*). The induction of phosphorylation of STING on S366 of IRF3 on S396 was inhibited in T404E compared to WT or T404A cells (Fig. 3*C*). We also found that IRF3 nuclear translocation (Fig. 3*D* and *SI Appendix*, Fig. S5*B*) and the induction of IRF3-dependent genes (Fig. 3*E*) were substantially inhibited in cells expressing T404E-STAT2. These results are consistent with preferential inhibition of these genes, mediated by STAT2 T404 phosphorylation in response to cGAMP and cdi-GMP in THP1 cells (*SI Appendix*, Fig. S5*C*). In summary, the phosphorylation of T404 of STAT2 increases its affinity for STING, which inhibits STING-dependent IRF3 activation, resulting in less IFN production.

How does the phosphorylation of STAT2 on T404 mediate its ability to inhibit the STING–IRF3 pathway? Our previous study (9) revealed that the Y245 phosphorylation of STING is a marker for late endosome trafficking and allows STING to form a tripartite complex with TBK1 and IRF3. We now find that cGAMP induces a higher level of Y245 phosphorylation in cells expressing WT or T404A-STAT2 than in cells expressing the T404E mutant (*SI Appendix*, Fig. S5*D*). These data reveal that STAT2 phosphorylated on T404 controls the trafficking of STING to late endosomes, limiting IRF3 activation and IFN production in response to cGAMP. Upon cGAMP treatment, IRF3 forms foci and colocalizes with STING and CD63, a marker of late endosomes (*SI Appendix*, Fig. S5*E*). Most importantly, we observed more STING–IRF3–CD63 foci, with a distinct pattern, in cells expressing T404A-STAT2 compared to cells expressing WT- or T404E-STAT2 (*SI Appendix*, Fig. S5*E*). Consequently, IRF3 nuclear translocation was inhibited in cells expressing T404E-STAT2 (*SI Appendix*, Fig. S5*F*). These data demonstrate that the phosphorylation of T404 drives the ability of STAT2 to inhibit STING by impeding STING trafficking from the ER.

### STAT2-Dependent Inhibition of STING Promotes Resistance to Chemotherapy and Tumor Progression.

Cancer cells frequently downregulate the expression and function of cGAS and STING, thus promoting malignancy (21, 35). Many studies have revealed the tumor-suppressive functions of these proteins in different types of cancer, including lung adenocarcinomas (*SI Appendix*, Fig. S6 *A* and *B*). Consistent with our new findings on the ability of STAT2 to inhibit STING-dependent signaling, there is a significant association between high levels of STAT2 in tumors and shorter overall survival in patients with lung adenocarcinomas (*SI Appendix*, Fig. S6*C*). Interestingly, in the same patient population, elevated expression of the IFN-I receptor subunit IFNAR1 correlated with better prognosis (*SI Appendix*, Fig. S6*D*), revealing an additional role of STAT2 that is independent of its tyrosine phosphorylation.

STING activation increases the kinase activity of TBK1 and IKKi (5, 6, 34). Interestingly, we found an inverse correlation between the expression of IKKi and TBK1 with patient prognosis in lung adenocarcinomas (*SI Appendix*, Fig. S6 *E* and *F*). IKKi is the dominant kinase for T404 phosphorylation (33), while TBK1, but not IKKi, is essential for IRF3 activation, which is critical for antitumor activity (21, 34, 35). Based on our finding that STAT2 preferentially inhibits the induction of IRF3-dependent genes, we propose that the IKKi-induced T404 phosphorylation of STAT2 mediates tumor progression in lung adenocarcinoma by inhibiting the tumor-suppressive function of the cGAS–STING pathway.

**Fig. 4.**
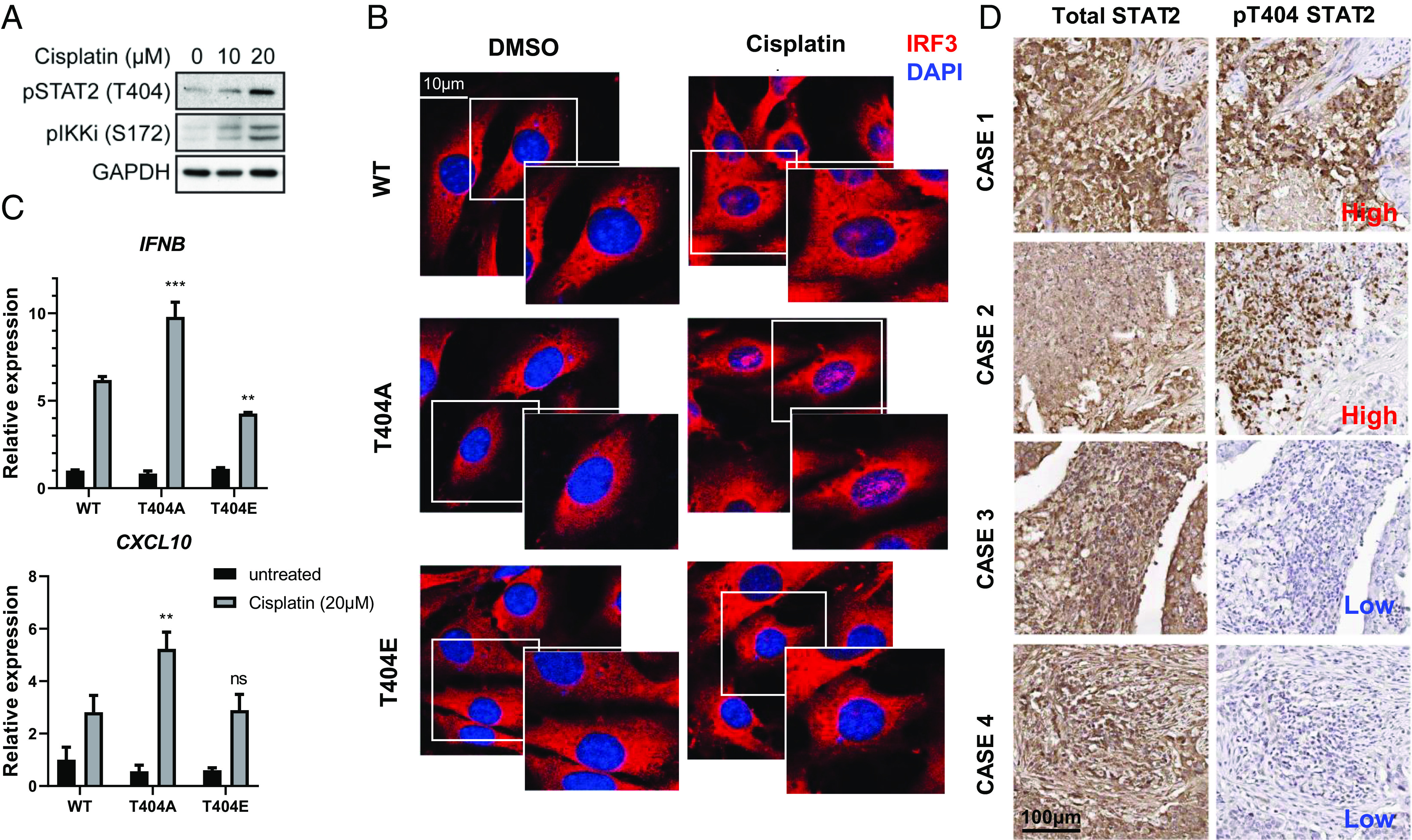
T404 phosphorylation of STAT2 promotes resistance to cisplatin in lung cancer cell lines and inhibits IRF3 nuclear translocation in response to cisplatin. (*A*) Calu-1 cells were treated with cisplatin for 24 h, and whole-cell lysates were analyzed by the Western method. (*B*) STAT2-deficient Calu-1 cells restored with WT, T404A, or T404E STAT2 were treated with cisplatin for 24 h. The cells were fixed then stained with IRF3 antibody (red) and DAPI for confocal analysis. (*C*) STAT2-deficient Calu-1 cells restored with WT, T404A, T404E, or Y690F STAT2 were treated with cisplatin (20 μM) for 24 h, followed by RT-PCR analysis. (*D*) Representative images of immunohistochemical staining for P-T404 STAT2 and STAT2 in specimens from human lung adenocarcinoma patients.

Sufficient activation of the cGAS–STING pathway is essential for the success of DNA-damage mediated therapy in cancer (21, 25). STING activation increases tumor cell death in response to DNA damaging agents, including cisplatin, a standard-of-care treatment for lung cancer (1, 36, 37). To investigate the role of STAT2 in resistance to cisplatin, we employed three lung cancer cell lines with different expression levels of STAT2: low in A549 cells and high in H196 and Calu-1 cells (*SI Appendix*, Fig. S7*A*). Increased expression of STAT2 attenuated cisplatin-induced cell death in A549 cells (*SI Appendix*, Fig. S7*B*), and downregulation of STAT2 expression increased cell death in Calu-1 and H196 cells (*SI Appendix*, Fig. S7 *C* and *D*). We found that cisplatin induces T404 phosphorylation (Fig. 4*A*), and the prevention of this phosphorylation by the T404A mutation promotes IRF3 nuclear translocation, which is opposite in the cells expressing the phospho-mimetic T404E mutation (Fig. 4*B*). Consequently, expression of T404A-STAT2 enhances the production of IRF3-dependent cytokines, such as IFN-β and CXCL10, which mediate potent immunogenic cell death (Fig. 4*C*) (38–40). To further evaluate whether the ability of STAT2 to mediate resistance to cisplatin depends on T404 phosphorylation, WT, T404A, T404E, or Y690F STAT2 were expressed in STAT2-deficient Calu-1 and H196 cells. The phosphorylation-deficient mutation T404A inhibited cisplatin resistance in Calu-1 cells, which have a high basal level of T404 phosphorylation when the WT protein is present (*SI Appendix*, Fig. S7*E*). In contrast, the GOF T404E mutation increased resistance to cisplatin in phosphorylation H196 cells, which have a low level of T404 phosphorylation when the WT protein is present (*SI Appendix*, Fig. S7*F*).

Using an antibody that specifically recognizes phosphorylated T404 (33), we analyzed T404 phosphorylation in a human tissue array that includes various types of lung cancer (Fig. 4*D* and *SI Appendix*, Fig. S8 *A* and *B*). T404 phosphorylation is readily detected in about half of these specimens, with about 20% of the samples showing a high level (*SI Appendix*, Fig. S8*C*). STING also connects genotoxic with immunogenic responses in tumor progression (41, 42). Activation of the STING pathway promotes the infiltration of T cells into tumors (43–45). Interestingly, about 20% of the human lung cancer specimens analyzed in *SI Appendix*, Fig. S8*D* showed noticeable infiltration of CD8+ cells. However, in tumors with a high level of T404 phosphorylation, only 7% of those had cytotoxic T cells (*SI Appendix*, Fig. S8*D*), revealing a trend toward a negative correlation between T404 phosphorylation and antitumor immunity (*P* = 0.12 by the chi-square test).

## Discussion

### Insight into STING-Dependent Signaling.

STING mediates three important innate immune functions: Inducing antiviral genes by activating IRF3, inducing inflammatory cytokines by activating NF-κB, and inducing autophagy to degrade intracellular pathogens or heterologous dsDNA (4, 46, 47). The binding of ligands permits STING to depart from the ER to different subcellular organelles and to generate the different signals noted above. Intracellular trafficking plays a crucial role in the complete activation of STING, including induction of IFNs, chemokines, and cytokines, and in initiating autophagy. Current studies have revealed two trafficking routes for STING: The first triggers the formation of autophagosomes by the translocation of STING to the ERGIC, allowing autophagy to clear cytosolic DNA and pathogens. In the second, STING departs from the ERGIC to the Golgi network and then to late endosomes, where it activates transcription factors, including IRF3, to induce the expression of genes encoding proteins important for defense against pathogens. These two routes will eventually introduce STING into autolysosomes, leading to its eventual degradation (4, 9).

The translocation of STING is regulated by posttranslational modifications and the interaction of STING with other proteins. For example, palmitoylation of C88 and C91 results in the translocation of STING to the Golgi apparatus, which is required for IRF3 activation (6). Furthermore, the K224R mutant of STING stays in the ER, thus failing to induce IFN production (48). Several proteins, including STIM1, NPC1, and SREBP2, influence trafficking by binding to STING, providing potential targets for future clinical research (11, 49). Our study demonstrates that STAT2 binds to STING and delays its trafficking, thus limiting the induction of IRF3-dependent genes, including *IFNB and CXCL10*. We show that STAT2 inhibits the phosphorylation of STING S366, which is required for the binding and subsequent activation of IRF3, but not NF-kB. Since STAT2 inhibits the transcription of IRF3-dependent, but not NF-kB–dependent genes, STAT2 may impair the ability of TBK1 to phosphorylate IRF3 by disrupting the IRF3–STING interaction. On the other hand, STAT2 has a strong nuclear export signal when Y690 is not phosphorylated (50). Therefore, in addition to its effect on IRF3 phosphorylation, if it binds to a STING–IRF3 complex, STAT2 may also inhibit the nuclear translocation of IRF3 and thus its transcriptional function.

Moreover, we reveal a unique negative feedback mechanism mediated by the phosphorylation of T404 of STAT2, induced by TBK1/IKKi following the binding of specific ligands to STING, resulting in less STING translocation to late endosomes. The STAT2–STING interaction reveals a crosstalk between cGAS–STING and STATs signaling that needs further investigation to reveal additional mechanistic details and therapeutic opportunities.

### Insight into Disease.

When cGAS binds to cytoplasmic DNA, it produces 2′, 3′-cGAMP which, in turn, activates STING. This pathway is a prominent topic in immuno-oncology research (51–53). In combination with immune checkpoint blockade, injection of irradiated tumor cells into mice promoted tumor clearance in a STING-dependent manner (54). In addition to enabling immune surveillance in the tumor microenvironment, STING agonists promote tumor regression by activating NLRP3 inflammasomes (55, 56). To avoid being recognized by STING-mediated innate immune responses, cancer cells frequently evolve to impede the cGAS–STING signaling pathway, including, but not limited to, downregulating the expression of key signaling proteins (57), limiting STING translocation to the Golgi (6, 46), and mutating the IFN-I locus (48). A recent study demonstrates that cGAS–STING drives IL-6–STAT3 signaling, which promotes the survival of chromosomally unstable cancers (26). Genotoxic stress induces IL-6 via STING-mediated activation of NF-κB, rather than IRF3. We find that high expression and T404 phosphorylation of STAT2 inhibit STING subcellular translocation and reduce the production of IRF3-dependent cytokines, including IFNs and T cell chemotaxis, without inhibiting IL-6 production, thus promoting tumor cell survival.

Since the function of STING in sensing pathogens and inducing antimicrobial innate immune responses is crucial, pathogens have evolved to evade host immunity by attenuating STING activation. Some proteins from pathogens specifically hinder STING trafficking, including M152 from Murine Cytomegalovirus (MCMV) (46) and IpaJ from *Shigella flexneri* (5, 58), Therefore, these proteins share a similar effect with STAT2 inhibition on STING activation, which affects only IRF3-dependent gene expression but not the expression of genes that depend on NF-κB activation. In addition to infectious diseases, gain-of-function (GOF) mutations of STING have been identified in STING-associated vasculopathy with onset in infancy (SAVI), as well as in patients with lupus (59–61). These mutations constitutively activate STING regardless of ligand binding (5). Evidence from genetic experiments in mice demonstrates that STING-associated lung diseases are independent of IRF3 but rely on T cells (62, 63). Wu et al. have shown that T cell cytopenia is due to disrupted calcium homeostasis caused by the constitutive ER departure of a GOF STING mutant (64). Even though IFN-I signaling is not thought to contribute to the onset of SAVI (62), Janus Kinase (JAK) 1/2 inhibitors inhibit STING signaling in vitro and in several patients carrying GOF STING mutations (65, 66) by an unknown mechanism. We find that JAK-induced tyrosine phosphorylation of STAT2 is not required for its ability to inhibit STING. Thus, we hypothesize that in addition to ameliorating interferonopathy, inhibiting JAKs could skew STAT2 away from ISGF3 to instead complex with STING and prevent its departure from the ER.

In summary, our study reveals that STAT2 is an ER retention factor that arrests STING trafficking. Further dissection of the mechanism underlying the maintenance of STING–STAT2 complexes, such as additional structural information, may inform the design of specific therapeutic approaches for balancing STING-mediated innate immune responses.

## Materials and Methods

### Cell Culture and Transfection.

Calu (HTB-54), A549 (CCL-185), H196 (NCI-H196), THP1 (TIB-202), L929 (CCL-1), HT1080 (CCL-121), HEK293T (CRL-11268), HeLa (CRM-CCL-2), and BJ cells (CRL-2522) were purchased from American Type Culture Collection (ATCC) (Vanassas, VA, USA), HT1080 STING^−/−^ cells were made by using Crispr/Cas9 sgRNA. U6A STAT2^−/−^ cells were previously described (67). The Barber lab graciously provided us with Primary STING^−/−^ Mouse Embryonic Fibroblasts (MEFs) (9, 48), while *SI Appendix*, Table S1 showcases a comprehensive list of the utilized cell lines. Plasmid transfections were conducted with Lipofectamine 2,000, following the manufacturer's protocol. In contrast, polyethyleneimine (PEI) was used for 2′, 3′-cGAMP transfections. To achieve this, 1 μg of 2′, 3′-cGAMP (final concentration: 8 μg/mL) and 2.25 μL of PEI (1 mg/mL, pH 7.0) were separately incubated in 0.25 mL of serum-free medium for 5 min. The two were then combined for 15 min before being added dropwise to the cells as previously described (9). HSV-1 strain KOS propagation and infection were performed as previously described (68, 69), with virus titers being determined via plaque assay.

### Western Analyses.

Following lysis in lysis buffer (50 mM Tris-HCl, pH 7.4, 150 mM NaCl, 5 mM EDTA, 1 mM DTT, 1 mM Phenylmethylsulfonyl Fluoride) and cocktail (Roche) at 4 °C for 15 min, the cells were centrifuged at 12,000× g at 4 °C for 10 min. The resulting cell lysates were either analyzed via SDS-PAGE or used for IP. After electrophoresis of the proteins in SDS-PAGE gels, they were transferred to Polyvinylidene Fluoride (PVDF) membranes (Bio-Rad). Subsequently, the membranes were immersed in 5% skim milk in Tris-buffered saline with Tween (TBST) buffer (150 mM NaCl; Tris, pH 7.4; and 0.1% Tween 20) for about an hour at room temperature and then left to incubate with the primary antibody in a cold room overnight. Western experiments were conducted with the designated antibodies and visualized through the use of Super-Signal West Pico Chemiluminescent substrate (Pierce Chemical), with the Western images being crafted with Adobe Illustrator.

### Immunoprecipitation.

For immunoprecipitations, cells were lysed in a buffer consisting of 20 mM HEPES, pH 7.5, 150 mM NaCl, 10 mM NaF, 1.5 mM MgCl2, 10 mM β-glycerophosphate, 2 mM EGTA, 1 mM Na_3_VO_4_, 1% (v/v) Triton X-100, 0.2% NP-40, and protease inhibitors (Roche Applied Science, Indianapolis, IN, USA). After centrifuging whole-cell lysates at 12,000× g for 10 min at 4 °C, the supernatant suspensions were used for immunoprecipitation, involving Protein A/G PLUS Agarose (Santa Cruz) and overnight incubation with 3 μg of mouse or rabbit monoclonal antibody at 4 °C. The related beads were washed six times with IP buffer before being heated in sodium dodecyl sulfate–polyacrylamide gel electrophoresis (SDS-PAGE) buffer for 10 min at 95 °C as previously described (9). The samples were then separated in SDS-PAGE gels and transferred onto PVDF membranes (Bio-Rad) for Western experiments performed with the designated antibodies and visualized through the use of Super-Signal West Pico Chemiluminescent substrate (Pierce Chemical).

### RT-PCR.

To extract cDNA, the RNeasy Mini Kit (Qiagen) was employed, and 0.5 ng of cDNA was utilized for Realtime PCR on a 384-well plate, using Applied Biosystem’s Power SYBR Green PCR mix in Roche LightCycler 480 II. The levels of induced mRNAs were standardized to 18S rRNA. The specificity of the primers was verified by analyzing the melting curves of the PCR products. The primer sequences used are provided in *SI Appendix*, Table S1.

### Immunofluorescence Staining and Confocal Microscopy.

Cells were cultured on 4-well slides and transfected with specified plasmids using lipo2000 for 24 h. The fixed cells were treated with 4% paraformaldehyde overnight at a low temperature and then permeabilized in Phosphate-Buffered Saline (PBS) containing 0.1% Triton X-100 for 10 min at room temperature. After being washed four times with PBS, the cells were blocked with 10% Bovine Serum Albumin (BSA) in PBS for 1.5 h at room temperature. The antibody was incubated with the cells overnight in a cold room, followed by three washes with PBS for 10 min. The cells were then treated with Goat anti-rabbit Alexa Fluor 594 (Invitrogen), anti-mouse Alexa Fluor 594 (Invitrogen), anti-rabbit Alexa Fluor 647 (Invitrogen), or Donkey Anti-Goat IgG H&L (Alexa Fluor® 488) (Thermo Fisher). Images were captured by confocal laser scanning microscopy (Leica True Confocal Scanning SP8) and processed with Leica LCS software as previously described (9). Fiji (ImageJ) software Co-localization plugin was utilized to identify protein colocalization, and Pearson’s colocalization coefficient was determined using Fiji software (ImageJ). Random images were collected for each condition, and higher parameters were set to generate clear results, resulting in fewer white spots for control and negative groups.

### Quantification and Statistical Analysis.

For qPCR analysis, the SD were calculated from three or more independent biological experiments and represented by error bars. Results from Western analyses, co-immunoprecipitations, and confocal experiments were considered representative of at least two independent experiments unless otherwise noted. The two-tailed *t* test using Prism 5 software (GraphPad) was used to determine statistical significance between two groups. All values represent means ± SD of the indicated independent experiments. NS, *P* > 0.05; *P < 0.05; **P < 0.01, ***P < 0.001.

## Supplementary Material

Appendix 01 (PDF)

## Data Availability

All study data are included in the article and/or *SI Appendix*.
