# Supplementary material for: STAT2 hinders STING intracellular trafficking and reshapes its activation in response to DNA damage
Source: Proc Natl Acad Sci U S A. 2023 Apr 10;120(16):e2216953120. doi: 10.1073/pnas.2216953120 (PMC10120020; doi:10.1073/pnas.2216953120)
Supplement: Supplementary file 1 — Appendix 01 (PDF) [file pnas.2216953120.sapp.pdf]

PNAS

Supporting Information for
STAT2 hinders STING intracellular trafficking and reshapes its activation in response to DNA damage

Chenyao Wang[1,5], Jing Nan[2], Elise Holvey-Bates[3], Xing Chen[1], Samantha Wightman[3], Muhammad-Bilal Latif[4], Junjie Zhao[1], Xiaoxia Li[1], Ganes C. Sen[1,*], George R. Stark[3,*] Yuxin Wang[3,*]

[1] Department of Inflammation and Immunity, Lerner Research Institute, The Cleveland Clinic Foundation, Cleveland, OH 44195, USA

[2] Institute of Cancer Biology and Drug Screening, School of Life Sciences, Lanzhou University, Lanzhou, Gansu 730000, China

[3] Department of Cancer Biology, Lerner Research Institute, The Cleveland Clinic Foundation, Cleveland, OH 44195, USA

[4] Pathology Advanced Translational Research Unit, Department of Pathology and Laboratory Medicine, School of Medicine, Emory University, Atlanta, 30322, GA, USA.

[5] Department of Medicine, Mayo Clinic College of Medicine and Science, Mayo Clinic, Rochester, MN 55905, USA.

Email: *Yuxin Wang (wangy7@ccf.org), George Stark (starkg@ccf.org), or Ganes Sen (seng@ccf.org)*

This PDF file includes:

Figures S1 to S8
Table S1

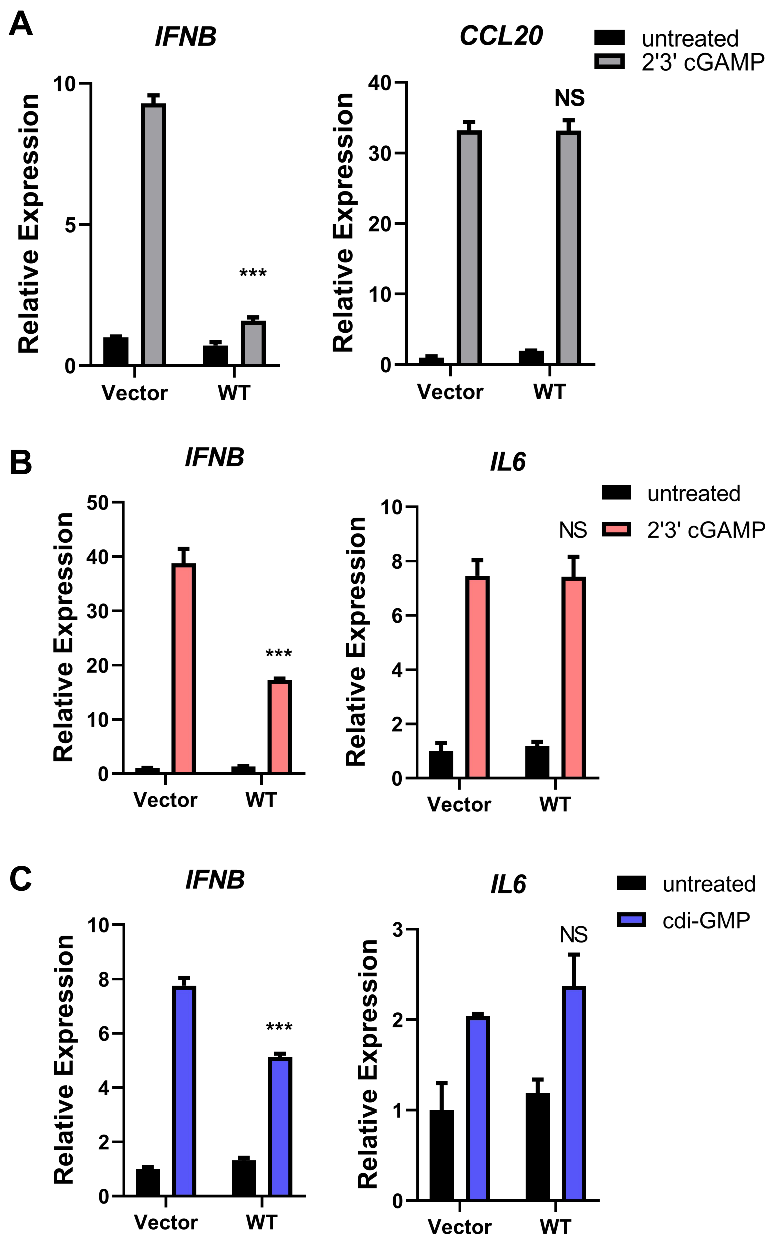

Fig. S1. High expression of STAT2 inhibits induction of IFNB in response to various STING agonists.

A. Calu cells over expressing vector (EV) or STAT2 (WT) were stimulated with 2'3'cGAMP (8 μg/ml) or cdi-GMP (10 μg/ml) (C) for 4 h, followed by RT-PCR analysis. NS: Not Significant, *** $P<0.001$ by two-way ANOVA.
B. THP1 cells over expressing vector (EV) or STAT2 (WT) were stimulated with 2'3'cGAMP (8 μg/ml) or cdi-GMP (10 μg/ml) (C) for 4 h, followed by RT-PCR analysis. NS: Not Significant, *** $P<0.001$ by two-way ANOVA.
C. THP1 cells over expressing vector (EV) or STAT2 (WT) were stimulated with cdi-GMP (10 μg/ml) or cdi-GMP (10 μg/ml) (C) for 4 h, followed by RT-PCR analysis. NS: Not Significant, *** $P<0.001$ by two-way ANOVA.

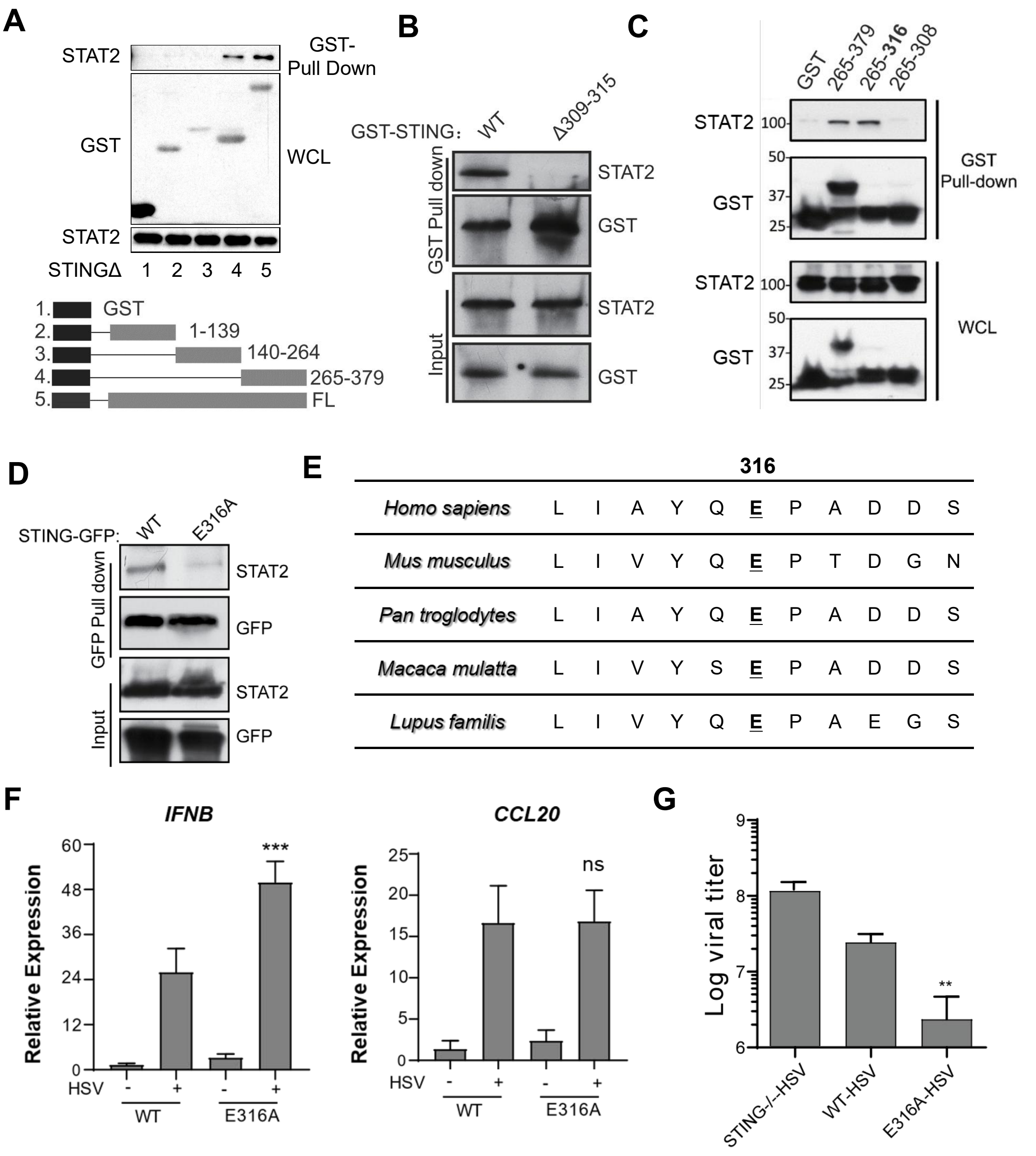

Fig. S2. Interaction with STING is essential for inhibition by STAT2

A. GST-pull-down assay, using HEK293T cells transfected with GST-tagged truncations of STING.
B. GST-pull-down assay, using HEK293T cells transfected with the indicated GST-tagged deletion of STING.
C. GST-pull-down assay, using HEK293T cells transfected with GST-tagged specific deletions of STING.
D. GST-pull-down assay, using HEK293T cells transfected with GST-tagged WT and E316A mutant of STING.
E. STING E316 and surrounding sequences across species.
F. STING-deficient HT1080 cells restored with WT- or E316A-STING were infected with HSV-1 (MOI=5) for 1 h, the media were refreshed for 4 h, followed by RT-PCR analysis.

G. STING-deficient HT1080 cells restored with WT- or E316A-STING were infected with HSV-1 (MOI=5) for 1h, then the media were refreshed for 16h. The media were analyzed for HSV by plaque assay.

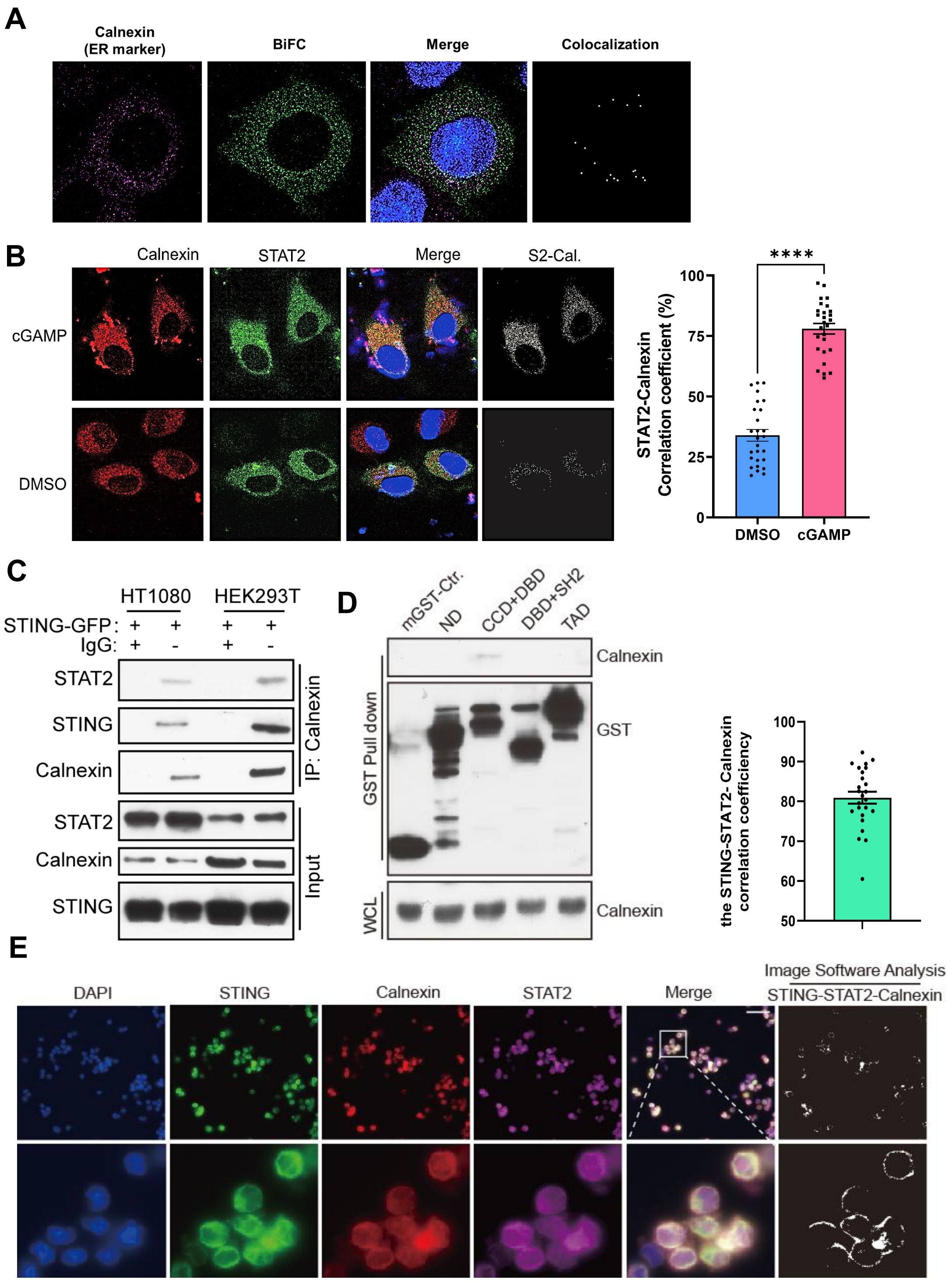

Fig. S3.

A. Calu-1 cells transfected transiently STAT2-STING-V155M BiFC were monitored by confocal fluorescence microscopy.
B. Calu-1 cells were transfected with STAT2-GFP (green) for 24 h, treated with cGAMP for 1 h. The cells were fixed and stained with calnexin antibody (red). Imaging data from confocal

fluorescence microscopy were analyzed by Fuji software to reveal co-localization, shown as white dots.

C. PBMC cells were cultured in RPMI-1640 medium and transfected with 8 μg/ml cGAMP for 1 h. The cells were fixed and then stained with the indicated antibodies and DAPI. Imaging data from confocal fluorescence microscopy were analyzed by Fuji software to reveal co-localization as white dots.
D. IP analysis of STAT2-STING-calnexin interactions, in HT1080 and 293T cells transfected with STING-GFP for 48 h and treated with 8 μg/ml cGAMP for 3 h.
E. GST-pull-down assay using HEK293T cells transfected with GST-tagged truncations of STAT2. ND: N-terminal Domain, CCD: Coil-coiled Domain, DBD: DNA-binding Domain, TAD: Trans-activation domain;

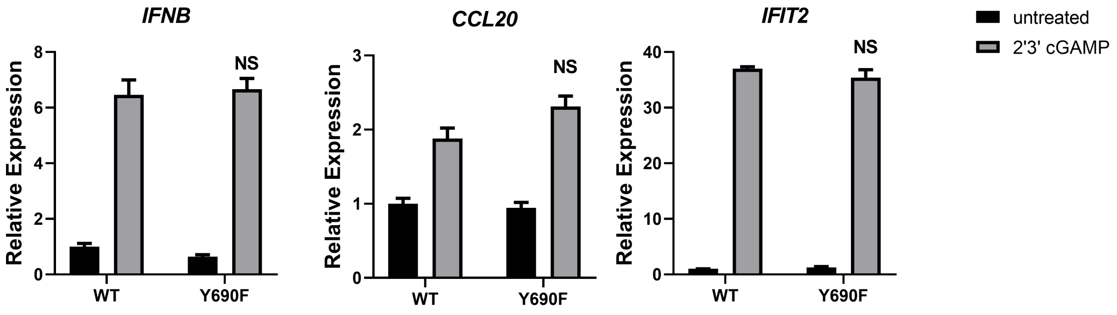

Fig. S4. STAT2 inhibits STING activation independently of Y690 phosphorylation

STAT2-deficient Calu-1 cells restored with WT STAT2 were stimulated with 2’3’cGAMP (8 μg/ml) for 4 h, followed by RT-PCR analysis.

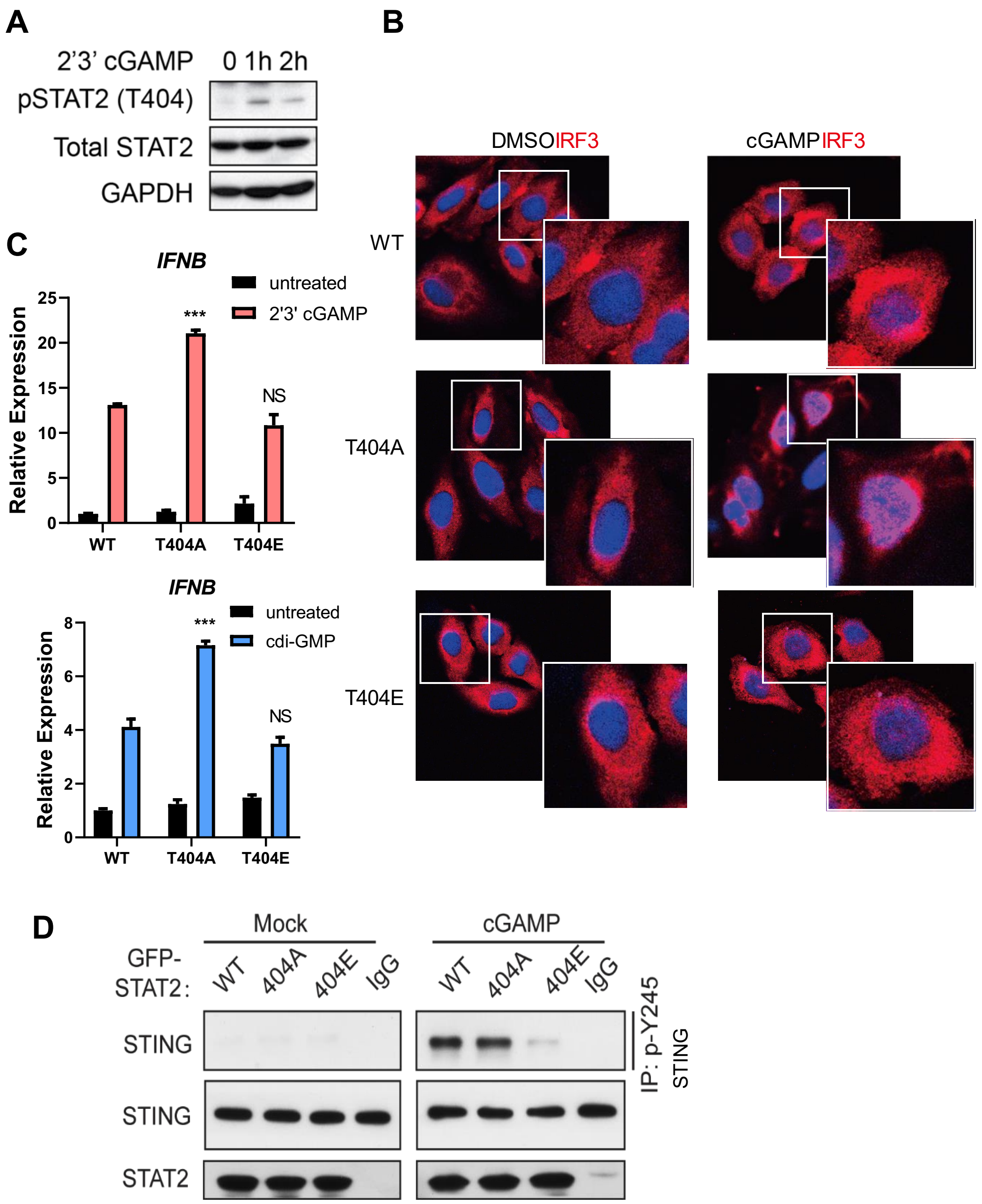

A
2'3' cGAMP
0 1h 2h
pSTAT2 (T404)
Total STAT2
GAPDH
B
DMSO IRF3
cGAMP IRF3
WT
T404A
T404E
C
IFNB
untreated
2'3' cGAMP
Relative Expression
***
NS
WT
T404A
T404E
IFNB
untreated
cdi-GMP
Relative Expression
***
NS
WT
T404A
T404E
D
Mock
cGAMP
GFP-
STAT2:
WT
404A
404E
IgG
STING
STING
STAT2
IP: p-Y245
STING

E

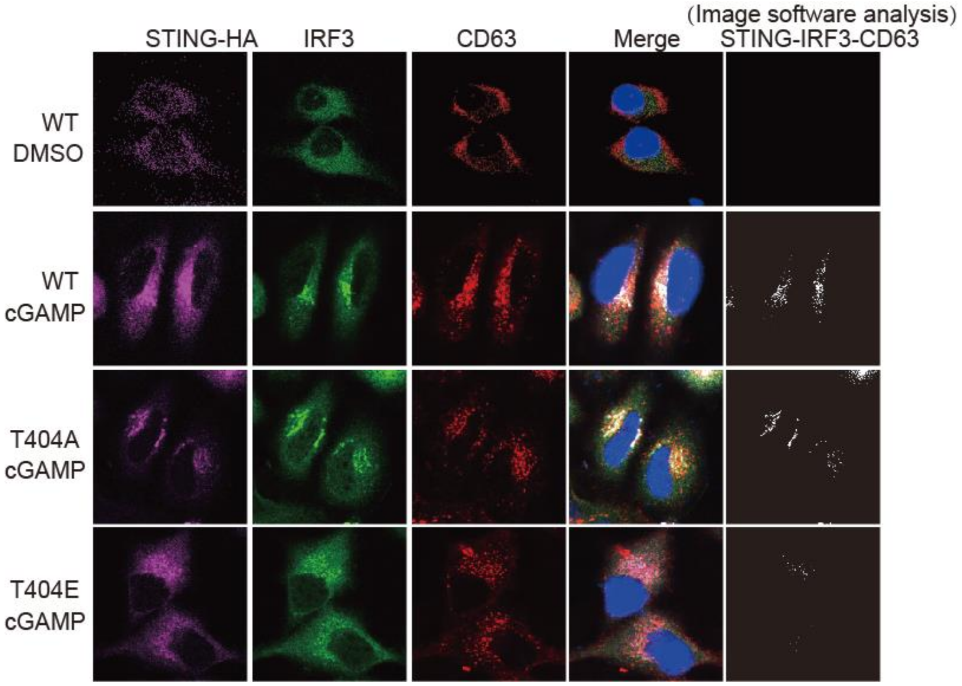

F

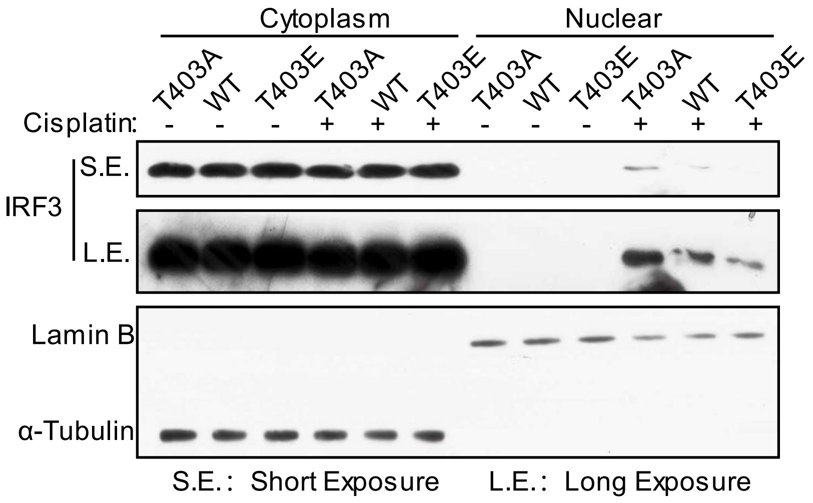

Fig. S5. STING mediates IRF3 activation on late endosomes.

A. Calu-1 cells were treated with cisplatin for 24 h, and whole-cell lysates were analyzed by the Western method.
B. The cells from Figure 3D were fixed, then stained with anti-IRF3 (red) and DAPI for confocal analysis.
C. THP-1 cells expressing WT, T404A, or T404E STAT2 were stimulated with 2'3'cGAMP (8 μg/ml) or cdi-GMP (10 μg/ml) for 4 h, followed by RT-PCR analysis.

D. STAT2-deficient Calu-1 cells restored with WT, T404A, or T404E STAT2 were stimulated with 2’3’cGAMP (8 µg/ml) for 3 h, followed by Western analysis
E. Primary STING$^{-/-}$ MEF cells were transfected with the indicated plasmids for 24 h and treated with cGAMP for 1 h. The cells were fixed, then stained with HA (purple), CD63 (red), and IRF3 (green) antibodies and DAPI. Imaging data from confocal fluorescence microscopy were analyzed with Fuji software to reveal co-localization as white dots. T403 is the residue corresponding to human T404 in mouse STAT2.
F. Primary MEF cells were transfected with the indicated plasmids for 24 h and treated with cGAMP for 1 h, followed by nuclear fractionation and analyzed by the Western method.

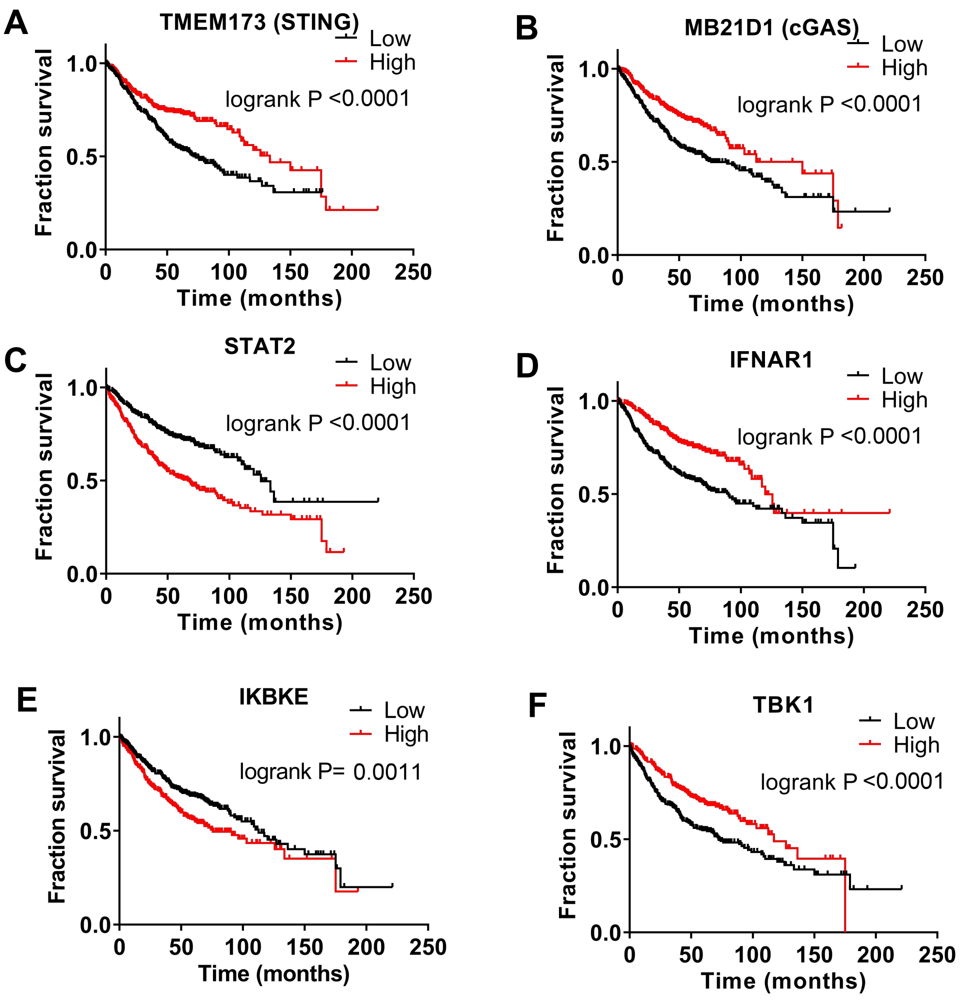

Fig. S6. Expression of STING-related genes affects prognosis of lung adenocarcinoma patients

Kaplan-Meier survival analysis of lung adenocarcinoma patients with respect to tumoral STING (A), cGAS (B), STAT2 (C), IFNAR1 (D), IKBKE (E), and TBK1 (F)

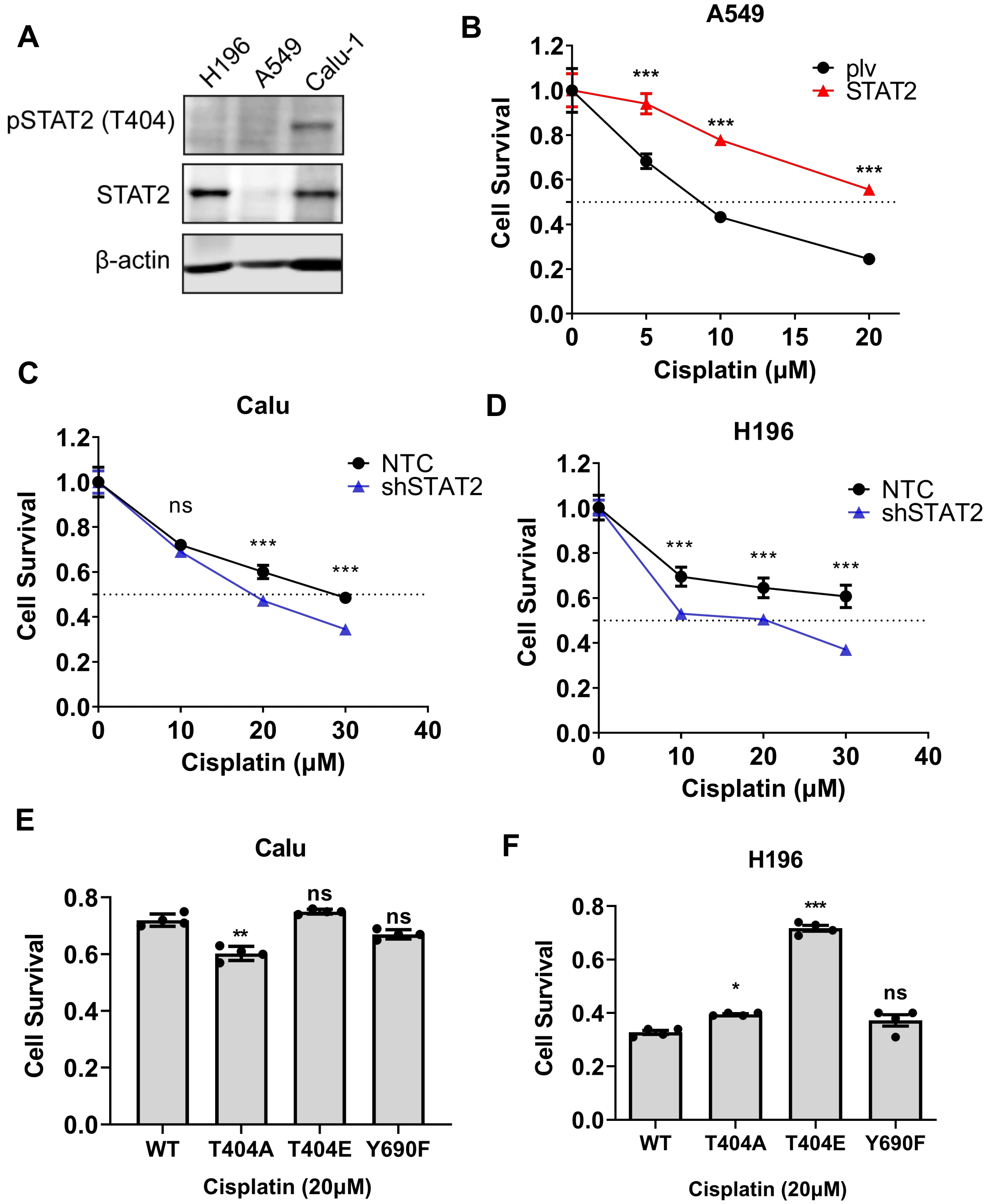

Fig. S7. pT404 of STAT2 promotes cisplatin-resistance in lung cancer cell lines.

A. Whole cell lysates of H196, A549, Calu-1 cells were analyzed by the Western method.
B. A549 cells expressing empty vector (plv) or STAT2 cDNA were treated with cisplatin for 72 h and cell survival was determined by using the MTT assay.
C. Calu-1 cells with or without STAT2 knockdown were treated with cisplatin for 72 h and cell survival was determined with the MTT assay.
D. H196 cells with or without STAT2 knockdown were treated with cisplatin for 72 h and cell survival was determined with the MTT assay.

E. STAT2-deficient Calu-1 cells restored with WT, T404A, T404E, or Y690F STAT2 were treated with cisplatin for 72 h and cell survival was determined with the MTT assay.
F. STAT2-deficient H196 cells restored with WT, T404A, T404E, or Y690F STAT2 were treated with cisplatin for 72 h and cell survival was determined with the MTT assay.

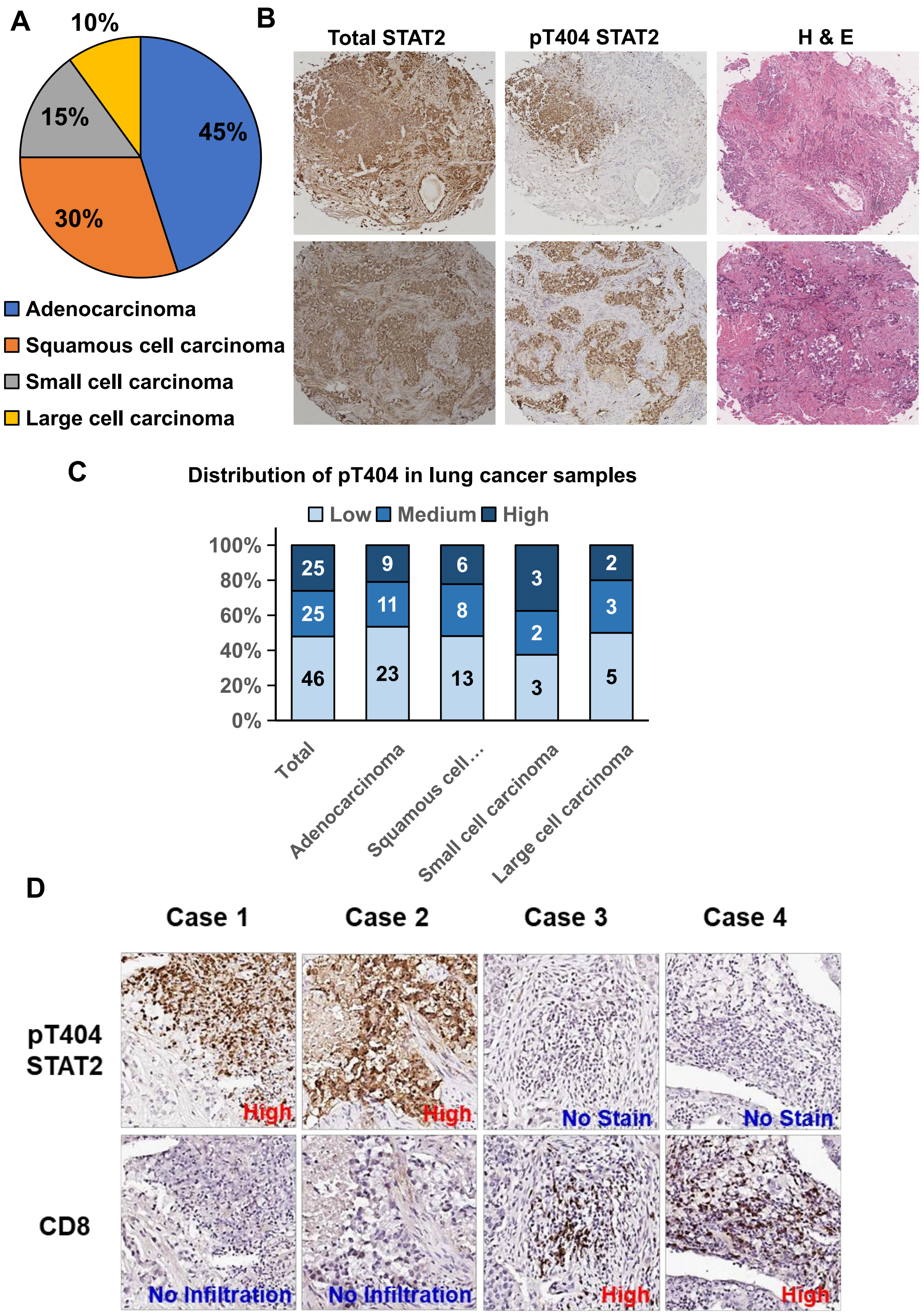

Fig.S8. T404 phosphorylation in lung cancer tissue arrays

A. A human tissue array that includes various types of lung cancer is shown.
B. Immunohistochemistry staining of human lung adenocarcinoma specimens. Representative staining of tumors high or low in phosphorylated T404 are shown.
C. Immunohistochemistry staining of human lung adenocarcinoma specimens. Distributions of staining of tumors high or low in phosphorylated T404 are shown.
D. Representative images of immunohistochemical staining for CD8 and phosphorylated T404 STAT2 in human lung adenocarcinoma specimens, showing an inverse correlation between P-T404 and CD8 infiltration.

**Reagent/Resource**	**Reference or Source**	**Identifier or Catalog Number**
**Experimental Models**		
C57BL/6J (M. musculus)	Wang et al, 2020	N/A
primary MEF STING-/- cells	Ni et al, 2017	N/A
U6A cells	Wang et al, 2020	N/A
HSV-1 (KOS)	Blaho et al, 2005	N/A
HT1080 STING-/-	This study	N/A
Human Mammary Epithelial Cells (HME)	Lonza	CC-2551
A549 cells	ATCC	Cat: CCL-185
HT1080 cells	ATCC	Cat: CCL-121
HEK293T cells	ATCC	Cat: CRL-11268
HeLa cells	ATCC	Cat: CRM-CCL-2
BJ cells	ATCC	Cat: CRL-4001
H196	ATCC	Cat: NCI-H196
Calu cells	ATCC	Cat: HTB-54
THP1 cells	ATCC	Cat:TIB-202
Human Peripheral Blood Mononuclear Cells (hPBMC)	Lonza	CC-2704
L929 cells	ATCC	Cat: CCL-1
DH5 alpha (E. coli)	ThermoFisher	Cat: 18265017
**Recombinant DNA**		
pLVX-human STAT2-GFP	This study	N/A
pLVX-human STAT2(T404A)-GFP	This study	N/A
pLVX-human STAT2(T404E)-GFP	This study	N/A
pLVX-human STING-GFP	This study	N/A
pGIPZ-human STAT2-UTR	This study	N/A
pLVX-mouse STAT2-myc	This study	N/A
pLVX-mouse STAT2(T403A)-myc	This study	N/A
pLVX-mouse STAT2(T403E)-myc	This study	N/A
mGST-STING(1-139)	This study	N/A
mGST-STING(140-264)	This study	N/A
mGST-STING(265-379)	This study	N/A
mGST-STING(Full length)	This study	N/A
Lenticrispr V2 hygro- SgSTING	This study	N/A
plVX-STING-VC83	This study	N/A
pLVX-STAT2-VN147	This study	N/A
mGST-STAT2(ND)	Wang et al, 2020	N/A
mGST-STAT2(CCD+DBD)	Wang et al, 2020	N/A
mGST-STAT2(DBD+SH2)	Wang et al, 2020	N/A
mGST-STAT2(TAD)	Wang et al, 2020	N/A
mouse STING-HA	This study	N/A

human STING-HA	This study	N/A
pLVX-STAT2-Flag	Wang et al, 2020	N/A
**Antibodies**		
p-T404 STAT2 (Rabbit host)	Wang et al, 2020	N/A
p-Y245 STING (Rabbit host)	Affinity Biosciences	Cat:#CF2P
HSV1 ICP0 (Mouse host)	Abcam	Cat: ab6513
Calreticulin(Rabbit host)	Cell Signaling technology	Cat:#12238
p-S366 hSTING (Rabbit host)	Cell Signaling technology	Cat:#19781
STAT2(Rabbit host)	Cell Signaling technology	Cat: #72604
p-S396 IRF3 (Rabbit host)	Cell Signaling technology	Cat:#29047
p-S172 IKKε (Rabbit host)	Cell Signaling technology	Cat:#8766
p-Y690 STAT2 (Rabbit host)	Cell Signaling technology	Cat:#88410
β-Tubulin (Rabbit host)	Cell Signaling technology	Cat:#2146
GAPDH (Rabbit host)	Cell Signaling technology	Cat:#5174
Actin (Rabbit host)	Cell Signaling technology	Cat:#3700
IRF3 (Rabbit host)	Abcam	Cat:ab68481
HA antibody(Mouse host)	Abcam	Cat:ab18181
STING antibody (Rabbit host)	Abclonal technology	Cat:A3575
Calnexin Antibody(Rabbit host)	Gensript	Cat: A01240
CD63(Mouse host)	MBL International Corporation	Cat:D263-3
IRF3 (Alexa Fluor® 488)	Abcam	Cat:ab204647
Human STING/TMEM173 Antibody	R&D system	AF6516
**Oligonucleotides and sequence-based reagents**		
h-18S rRNA-F, CTACCACATCCAAGGAAGCA	This study	Material and Method section
h-18S rRNA-R,TTTTTCGTCACTACCTCCCCG	This study	Material and Method section
h-IFNβ-F, CAACTTGCTTGGATTCCTACAAAG	This study	Material and Method section
h-IFNβ-R , TATTCAAGCCTCCCATTCAATTG	This study	Material and Method section
h-IFNα-F, AATGACAGAATTCATGAAAGCGT	This study	Material and Method section
h-IFNα-R, GGAGGTTGTCAGAGCAGA	This study	Material and Method section
h-Ccl20-F, AAGTTGTCTGTGTGCGCAAATCC	This study	Material and Method section
h-Ccl20-R, CCATTCCAGAAAAGCCACAGTTTT	This study	Material and Method section
h-Ifit2-F,TGCACTGCAACCATGAGTGAGAACA	This study	Material and Method section
h-Ifit2-R,GCCAGTAGGTTGCACATTGTGGC	This study	Material and Method section
h-GAPDH-F, TCCCTGAGCTGAACGGGAAG	This study	Material and Method section
h-GAPDH-R, GGAGGAGTGGGTGTCGCTGT	This study	Material and Method section
h-IL6-F, AGACAGCCACTCACCTCTTCAG	This study	Material and Method section

h-IL6-R, TTCTGCCAGTGCCTCTTTGCTG	This study	Material and Method section
h-CXCL10-F, GGTGAGAAGAGATGTCTGAATCC	This study	Material and Method section
h-CXCL10-R, GTCCATCCTTGGAAGCACTGCA	This study	Material and Method section
HSV-ICP0-F , GTCGCCTTACGTGAACAAGAC	This study	Material and Method section
HSV-ICP0-R , GTCGCCATGTTTCCCGTCTG	This study	Material and Method section
Si-mSTAT2	Santa Cruz Biotechnology	sc-37272-PR
SgSTING sequence :GCTGGGACTGCTGTTAAACG	This study	Material and Method section
**Chemicals, enzymes and other reagents**		
2'3'-cGAMP	InvivoGen	Cat:tlrl-nacga23-02
Cisplatin	Selleckchem	Cat:NSC 119875
Polyethyleneimine (PEI)	Mpbio	Cat:195444
Lipofectamine 2000	Invitrogen	Cat:11668019
GFP trap	Chromotek	Cat: gta-10
IFN-β	PBL Interferon Source	Cat:11415-1
**Software**		
Image J	https://imagej.net/Fiji	
	Schindelin, J.; Arganda-Carreras, I. & Frise, E. et al. (2012),	
GraphPad Prism version 8.3 for Windows, GraphPad Software	www.graphpad.com	
**Other**		
Kits, instrumentation, laboratory equipment, lab ware etc. that are critical for the experimental procedure and do not fit in any of the above categories can be listed here.		
RNeasy Mini Kit	Qiagen	Cat: 74104
Multiple lung cancer with lung tissue array	Biomax	Cat: LC1201a

Table S1
